# FHL3 Contributes to EMT and Chemotherapy Resistance Through Up-Regulation of Slug and Activation of TGF*β*/Smad-Independent Pathways in Gastric Cancer

**DOI:** 10.3389/fonc.2021.649029

**Published:** 2021-06-04

**Authors:** Guodong Cao, Pengping Li, Xiaobo He, Mengyao Jin, Mengying Li, Sihan Chen, Xin Xu, Qiang Sun, Maoming Xiong, Bo Chen

**Affiliations:** ^1^ Department of General Surgery, The First Affiliated Hospital of Anhui Medical University, Hefei, China; ^2^ Department of Breast Surgery, The First People’s Hospital of Xiaoshan District, Hangzhou, China; ^3^ Department of Clinical Medicine, Anhui Medical University, Hefei, China; ^4^ Jiangsu Key Laboratory of Biological Cancer, Cancer Institute, Xuzhou Medical University, Xuzhou, China

**Keywords:** epithelial to mesenchymal transition, chemotherapy resistance, FHL3, ubiquitination, gastric cancer

## Abstract

**Background:**

Gastric cancer presents high risk of metastasis and chemotherapy resistance. Hence, it is important to understand the mechanisms of gastric cancer distant metastasis and chemotherapeutic resistance. Our previous study has revealed Four and a Half LIM Domains 3 (FHL3) plays as a binding partner of Glycogen Synthase Kinase 3 Beta (GSK3*β*), promoted tumor metastasis in pancreatic cancer. However, the role of FHL3 in gastric cancer still remains unclear.

**Methods:**

TCGA database and clinical samples are used for exploring the role of FHL3 in disease progression and prognosis. Oxaliplatin (OHP) resistance cell lines were established to study the role of FHL3 in chemotherapy resistance. The experiments about cell proliferation, apoptosis, and metastasis were performed to measure the chemotherapy effects of sh-FHL3 on gastric cancer cell lines and *in vivo*. That FHL3 changed the EMT phenotype was verified by western blot. Finally, we explored the mechanism of FHL3-mediated EMT and chemotherapy resistance.

**Results:**

mRNA and protein level of FHL3 were significantly up-regulated in gastric cancer tissues when compared with adjacent tissue. FHL3 higher expression is always accompanied with higher TNM stage and worse overall survival. FHL3 over-expressed could lead to OHP resistance. Knockdown of FHL3 slightly inhibited the cell growth, while it obviously sensitized the chemotherapy *in vivo* and *in vitro*. In addition, down-regulation of FHL3 increased the mesenchymal markers, such as Slug, Snail, Twist Family BHLH Transcription Factor 1 (Twist1), and Vimentin, while it decreased the epithelial marker E-cadherin. Cell and animal experiments also proved that down-regulation of FHL3 can decrease cancer cell metastasis. For mechanism study, FHL3 knockdown down-regulated the expression level of Mitogen-Activated Protein Kinase (MAPK)/Extracellular Regulated Protein Kinase (ERK) pathway and Transforming Growth Factor-*β* (TGF*β*)/Phosphatidylinositol 3-Kinase (PI3K)/protein kinase B(Akt)/GSK3*β*-(Ring Finger Protein 146) RNF146/ubiquitin pathway. FHL3 competitively bonded the ubiquitin complex (Slug/GSK3β/RNF146) with Slug and inhibited ubiquitination of Slug. Mesenchymal phenotype cells hold higher level of Multidrug Resistance Gene1 (MDR1), and the FHL3 knockdown reverts the MDR1 in this type cell.

**Conclusion:**

FHL3 high expression contributed to EMT and chemotherapy resistance *via* MAPK, and PI3K pathways were activated. FHL3 competitively bonded the ubiquitin complex with Slug, resulting in the up-regulation of Slug and leading to metastasis of gastric cancer.

## Introduction

Gastric cancer (GC) is the fifth most common malignancy and accounts for the third leading cause of cancer death (1). Although prediction about the prognosis of GC is mainly according to the lymph node and distant metastasis status, increasing lines of evidence show that conventional staging criteria fail to sufficiently distinguish the prognostic differences of GC (2). Due to the high risk of lymph node metastasis and distant metastasis, about 65% cases of GC appear to have tumor recurrence and disease progression after surgical treatment, which leads to <35% rate of 5-year survival of GC (3). Many patients with clinical indications (advanced TNM stage) have to receive chemotherapy after resection surgery. As for the first-line chemotherapy drug for GC, 5-FU and oxaliplatin (OHP) usually appear tolerant and resistant during treatment; chemotherapy resistance is the main barrier that leads to unsatisfactory clinical outcomes (4). Thus, new prognostic biomarkers for GC are urgently needed to improve the early diagnosis and chemotherapy effectiveness of gastric cancer. Meantime, the mechanistic understanding of tumor metastasis and chemotherapy resistance should be improved to develop novel targets for advanced gastric cancer.

As a widely recognized mechanism, epithelial–mesenchymal transition (EMT) plays an irreplaceable role in cancer metastasis and invasion. As we all know, reduction of E-cadherin is a symbol of EMT process (5–7). Besides, down-regulation of other epithelial genes, such as Zonula Occludens Gene-1 (ZO-1) and occludin, and the up-regulation of mesenchymal genes, such as N-cadherin, Vimentin, *α*-Smooth Muscle Actin (*α*-SMA), and fibronectin, are the distinctive changes in the EMT (5–8). EMT-associated transcriptional factors (EMT-TFs), including Slug, Snail, Twist, and Zinc Finger E-Box Binding Homeobox (Zeb), directly regulate the expression of E-cadherin by binding to the promotor sequence of E-cadherin, which is named E-box (5–8). Most signaling pathways interfere with EMT process *via* the regulation of E-cadherin through EMT-TFs, such as Smad-independent TGF*β* pathway and Smad-dependent TGF*β* pathway (9–11). For the former one, TGF*β* can initiate the Wnt/*β*-catenin pathway, PI3K/Akt pathway, and MAPK/ERK pathways to up-regulate the level of EMT-TFs (11). For the later one, TGF-*β* activates Smad-complex/EMT-TFs to up-regulate the level of EMT-TFs (9).

Interestingly, recent studies implied that the roles of EMT beyond the traditional views in which it was the pivotal role in initiation of tumor metastasis and invasion and developing lines of evidence pointed out that the EMT-associated components (such as Snail, zeb, and Twist1) are closely related to drug resistance (12, 13). And, the mechanism of EMT-associated chemotherapy is complex. Cancer stem cell (CSC) is a feature in mesenchymal phenotype tumor cells, which is closely related to chemotherapy resistance (14–16). In addition, some regulating factors directly related to EMT can promote chemotherapy resistance. EMT-TFs including Twist, Snail, Slug, and Forkhead Box C2 (FOXC2), regulate chemoresistance by increasing the expression of ATP-binding cassette (ABC) transporters in breast cancer, which is important for chemoresistance (17, 18). Slug can inhibit the activity of caspase-9, leading to chemoresistance of tumor (19). However, single inhibition of EMT-TFs has few effects in reverting chemoresistance. The main reason of this phenomenon is other pathways which participate in the regulation of EMT process. According to previous studies, some EMT-associated pathways are also important in chemoresistance mechanism, such as PI3K/Akt pathway, MAPK pathway, and hypoxia pathway, through up-regulation of ABC transporters and decreasing apoptosis (20–25). Thus, the mechanism of EMT-associated chemotherapy resistance is complicated and unclear; more work is needed to figure out the mechanism of EMT-associated chemotherapy resistance.

LIM-only protein Four-and-a-Half LIM domain (FHLs), including FHL1, FHL2, and FHL3, are characterized by evolutionarily conserved LIM domains and one conserved LIM superfamily domain (26). FHLs are reported as the transcriptional factors, which participate in lots of signaling pathways. Simply, FHL1–2 are reported in the regulation of TGF*β*/Smad-independent pathway, such as PI3K/Akt, Wnt/*β*-catenin, and MAPK/ERK pathways. FHLs participate in the EMT process and chemo-radio-therapy resistance in pancreatic cancer, breast cancer, and osteosarcoma (7, 26, 27). Besides, FHL1–3 are considered as inhibitors of cell cycle checkpoints Cell Division Cycle 25 (CDC25) in pancreatic cancer and HeLa cell line and that they always lead to radioresistance (28). In addition, some studies show that FHLs can interact with Estrogen Receptor-*α*Polypeptide (ER-*α*) to make tumor progression in breast cancer (29, 30). Another study shows FHL2 can directly interact with epithelial phenotype marker ZO-1 to promote tumor invasion in breast cancer (31). In our previous study, FHL3 also acts as a regulator in ubiquitin degradation process of EMT-TFs through Akt/GSK3*β*/ubiquitin pathway in pancreatic cancer (7). However, some previous studies suggest that FHL1–3 perform as a tumor repressor in lung cancer, liver cancer, and breast cancer. As of now, no research article has revealed the role of FHL3 in gastric cancer, especially the regulatory mechanism of FHL3 in chemoresistance and metastasis.

In fact, our previous study has proved that FHL3 is an important role in the regulation of EMT. In this study, we explored the potential relationship between FHL3 and EMT/chemotherapy resistance in gastric cancer. Simply, we explored the role of FHL3 in disease progression and overall prognosis by investigating TCGA database and clinical GC samples. Oxaliplatin (OHP) resistance cell lines were established to study the role of FHL3 in chemotherapy resistance. The experiments about cell proliferation, apoptosis, and metastasis were performed to measure the chemotherapy effects of sh-FHL3 on gastric cancer cell lines and *in vivo*. That FHL3 changed the EMT phenotype was verified by western blot. The mechanism of FHL3-mediated EMT and chemotherapy resistance was clarified.

## Methods and Materials

### Gastric Cancer Sample Preparation

This study was approved by The First Affiliated Hospital of Anhui Medical University Review Board and the ethics committees of Anhui Medical University. Patients gave their informed consent to use gastric cancer samples and slices in this study. 120 matched paraffin-embedded tumor tissue sections and 16 paired fresh frozen tissues were collected. All patients underwent total or partial gastrectomy at the First Affiliated Hospital of Anhui Medical University from 2013 to 2016. All patients with gastric cancer were confirmed by at least two pathologists. Follow-up time was estimated from the date of surgical treatment to that of an event (*i.e.*, patient death or tumor recurrence) or withdrawal.

### Bioinformatic Analysis

Gene profiles of GC and non-tumor adjacent tissues based on microarray were downloaded from the Gene Expression Omnibus database (GEO; https://www.ncbi.nlm.nih.gov/geo/). TCGA and GTEx RNA sequencing FPKM data of GC, non-tumor adjacent tissues, and normal stomach tissues were downloaded from the UCSC Xena database (https://xenabrowser.net/hub/). The proteomic data of GC and non-tumor adjacent tissues were downloaded from PRIDE Archive under the accession number PXD011821. KEGG pathway analysis was performed using R clusterProfiler package. Gene Set Enrichment Analysis (GSEA) was performed by the JAVA program using gene set collection (c2.cp.v7.1.symbols.gmt) from the MsigDB.

### Cell Culture and OHP-Resistance Cell Lines

Gastric cancer cell lines (SGC-7901, HGC, AGS, and N87) and normal gastric epithelial cells (GES-1) were obtained from the cell bank of the Chinese Academy of Science. All cell lines were cultured in RPMI-1640 medium (Gibco, USA). All culture media were supplemented with 10% fetal calf serum and 100 units/ml penicillin and streptomycin. Gastric cancer cell lines (HGC and SGC) are firstly detected by the IC50 of OHP. Secondly, the cells are screened in this dose-treatment of OHP for at least three generations to establish the OHP-resistance cell lines. Then, the OHP IC50 of those cells is re-detected, and the cells are screened in the this IC50 OHP again. The screen cycle is performed at least six times.

### Western Blot Analysis

Total protein extraction: Cells were harvested with a cytology brush, lysed with RIPA lysis buffer (Sigma-Aldrich, USA) supplemented with a phosphorylase and protease inhibitor mixture (Thermo Fisher Scientific, USA), and quantified by a BCA assay.

Total protein was extracted from the normal mucosa, para-carcinoma, and corresponding tumor tissues of 10 GC patients using RIPA lysis buffer (Beyotime, Shanghai, China). The protein concentration was quantified using the Enhanced BCA Protein Assay Kit. The equivalent proteins in each pair of specimens were separated by SDS–PAGE on 12% polyacrylamide gels and electrotransferred to polyvinylidene fluoride membranes. After blocking in TBST (Tris buffered saline/Tween-20 buffer) containing 5% skim milk for 1 h at room temperature, the membrane was incubated in TBST solution containing anti-FHL3, anti-E-cad, anti-ZO1, anti-Vimentin, anti-Twist1, anti-snail1, anti-Slug, anti-ERK, anti-p-ERK, anti-p38, anti-p-p38, anti-JNK, anti-p-JNK, anti-PI3K, anti-p-PI3K, anti-Akt, anti-p-Akt, anti-GSK3*β*, anti-p-GSK3*β*, anti-TGF*β*, anti-Smad2/3, anti-p-Smad2/3, anti-Smad4, anti-p-Smad4 (1:1,000, Abcam, Cambridge, UK) overnight at 4°C. After washing three times in TBST, the membranes were incubated with the corresponding secondary antibodies in TBST along with 3% skim milk powder for 1 h at room temperature. After three washing steps in TBST, the band intensity was measured using the BandScan software. The protein bands were normalized to GAPDH signals. Western blot bands were quantified with ImageJ software (NIH, USA).

### MTT Assay

The cells in the logarithmic phase were plated onto 96-well plates at a density of 5,000 cells per well in 200 µl of culture medium and incubated for 24, 48, and 96 h at 37°C with 5% CO_2_. A volume of 20 µl MTT solution (5 mg/ml; Solarbio Science & Technology, Beijing, China) was added into each well and incubated for another 4 h. The MTT solution was then removed, and 100 µl dimethyl sulfoxide (Sigma) was added to each well. The relative optical density (OD) was measured at 570 nm (Spectra Max, USA), and the experiment was repeated three times.

### Immunofluorescence

Briefly, 2.5 × 10^4^ cells were seeded in 24-well plates for 24 h, fixed with 4% paraformaldehyde, permeabilized with 0.5% Triton X-100 and blocked with 5% BSA (Sigma-Aldrich) for 1 h at 37°C. The samples were incubated with a primary antibody (FHL3, 1:200, Proteintech) overnight at 4°C. Subsequently, the cells were washed with PBS and incubated with secondary antibodies for 1 h at room temperature before being washed again. Finally, the nuclei were stained with 15 μl DAPI (Sigma-Aldrich, USA) before detection with a fluorescence microscope (Carl Zeiss, Germany).

### Colony Forming Efficiency Assay

Firstly, cells were seeded in six-well plates at a density of 1,500 cells per well and incubated in 37°C for 10 days. Then cells were washed with PBS and fixed with 1 ml 4% formaldehyde solution. Then 1 ml crystal violet staining solution was added and washed with PBS for three times after 30 min. The newly formed colony units were counted by summing the number of different fields (100× microscape, five fields).

### Live and Death Staining

Cells (10 × 10^4^/per well) were seeded in a six-well plate, and then the cells were transfected with three FHL3 siRNA for 48 h. After that, AO and PI solutions (1% AO and PI, 5 ul AO and 5 ul were added into dyeing diluent buffer) were added into plates and maintained for 0.5 h before observing by an inverted fluorescent microscope. AO could stain the live cells that show the green fluorescence; PI could stain the dead cells that show the red fluorescence. Fluorescence intensity was quantified by ImageJ.

### Wound-Healing Assay

Cells (40 × 10^4^/per well) were cultured in a six-well plate and grown to 90% confluence in 2 ml of culture medium. A 200 µl plastic tip was used to create an artificial wound. Images were taken at 0, 24, and 48 h after scratching. The cell mobility = (0 h width- the indicated time points width)/0 h width × 100%.

### Migration Assay

Transwell (Corning Life Sciences, Bedford, MA, USA) was used to assess GC cell migration. For migration assays, 1 × 10^5^ cells were added to 200 µl serum-free DMEM in the upper chamber, and the lower chamber was filled with 600 µl culture medium. After incubation at 37°C in an atmosphere containing 5% CO_2_ for 24 h, the non-migrated cells were carefully removed with a wet cotton swab. Finally, the cells were stained with Giemsa (Sigma, USA) for 10 min followed by imaging and counting under an inverted microscope (100× magnification).

### Animal Experiments Protocols

#### Breeding Conditions and Method of Euthanization

Male nude mice and severe combined immunodeficiency (SCID) mice were bred in the SPF condition, 26–28°C, 40% humidity, 10 h illumination. When the *in vivo* experiments were performed, the nude mice were killed by cervical dislocation.

### Subcutaneous Tumor Model

All animal procedures were performed in accordance with the Guidelines for Care and Use of Laboratory Animals of Anhui Medical University and approved by the Animal Ethics Committee of Anhui Medical University.

Male nude mice (4 to 6 weeks old) obtained from the SLAC (Shanghai, China) were randomly divided into two groups (three nude mice per group). A total of 1 × 10^6^ cells (FHL3-NC and FHL3-SH1 cells) in 100 µl PBS were injected subcutaneously. 4 weeks later, the mother tumors are harvested to make the same volume transplanted tumors. Then the FHL3-NC-derived and FHL3-SH1-derived tumors are transplanted into mice of different groups (five nude mice per group). The tumor volume is investigated every day. All mice were sacrificed, and the tumors are harvested to determine the tumor volume (MaA MiA^2^/2; MaA = Major axis, MiA = Minor axis), followed by processing into sections for HE staining and Ki67 staining.

### Orthotopic Transplantation Model

Simply, the transplanted tumors are transplanted into the subserosa of the stomach in SCID mice. 4 weeks later, mice were executed to investigate the volume and weight of tumors.

### Lung Metastasis Model

Simply, 1 × 10^6^ cells are injected into the tail vein of SCID mice after 4 weeks, and the lungs are harvested for frozen sections to investigate the metastatic tumors by FITC-tag.

### Immunoprecipitation and Recombination Plasmid System

Primers of Slug, FHL3, and RNF146 are inserted into plasmid pcDNA 3.1(−) (Addgene). Briefly, cDNA templates are synthesized through PrimeScript RT Reagent Kit (TaKaRa, China); CDS of genes are amplified with PrimeSTAR^®^ GXL DNA Polymerase (TaKaRa, China); products are purified by SanPrep Column DNA Gel Extraction Kit (Sangon Biotech, China); the purified products and plasmids are treated with restriction endonuclease (Xho1, EcoR5, and Xba1 come from NEB, USA) respectively; recombination of plasmids are performed through homologous recombination with Hieff CloneTM Plus One Step Cloning Kit (Yeasen Biotech, China). Cells were transplanted into six-well plates for 24 h followed by transfection of RP for different times with Hieff TransTM Liposomal Transfection Reagent (Yeasen Biotech, China) to find out the best transfection efficiency, according to the manufacturer’s instructions.

A total of 1 × 10^7^ cells were harvested with a cytology brush and lysed with RIPA lysis buffer (Yeasen Biotech, 20118ES60) to isolate the protein supernatant, followed by adding magnetic beads (Anti-Myc, Anti-HA Bimake and Anti-Flag) with continuous slight mixing at 4°C for 24 h. Then, the magnetic beads were isolated with a magnet (Bimake), followed by washing with TBS. Finally, the products were boiled before being dissolved in 5× SDS (Yeasen) for 5–10 min for Western blot assays.

### Statistics

All independent cell experiments were repeated three times. All experimental data are presented as the mean ± SD. Statistical Package for the Social Sciences version 21.0 (SPSS Inc., USA) was used for the statistical analyses. ANOVA, paired t-test, Chi-square () test, and a non-parametric test (Mann–Whitney U) were used for statistical analysis in different situations. Statistical significance was defined as *P <*0.05 (*P < 0.05; **P < 0.01; ***P < 0.001). All histograms and curves were constructed with GraphPad Prism 6 software (GraphPad Software, La Jolla, CA, USA).

## Results

### FHL3 Plays a Disease Progression Role and Prognosis Prediction Biomarker in GC

**Figure 1:**
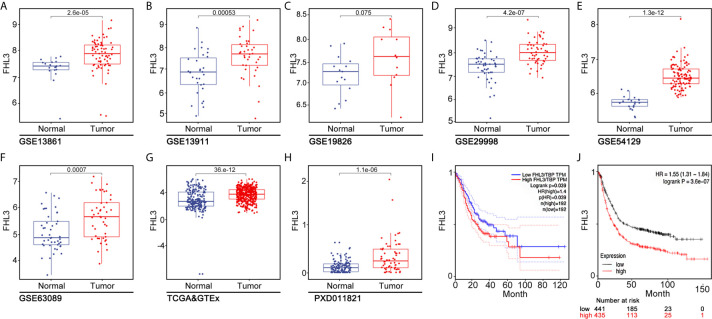
FHL3 is an independent risk factor in gastric cancer. (1) The expression level of FHL3 in gastric cancer and its adjacent tissues was analyzed in TCGA database, GTEx, and Beijing dataset **(A–H)**; the results suggested that FHL3 is highly expressed in tumor tissues. (2) The relationship between FHL3 expression level and prognosis is performed by Kaplan–Meier analysis in TCGA, KM-plotter cohorts; the results suggested that FHL3 is a prognostic indicator in gastric cancer; FHL3 overexpression always means poor overall survival **(I, J)**.

To investigate the clinical relevance of FHL3 in GC, we systematically analyze multiple publicly available gene expression datasets [The Cancer Genome Atlas/Genotype Tissue Expression (TCGA/GTEx), GSE13861, GSE13911, GSE19826, GSE29998, GSE54129, and GSE63089], which contain >1,000 gastric cancer patients. We noticed that FHL3 mRNA expression is remarkably up-regulated in GC (**Figures 1A, B, D–G**, *P* < 0.001). In line with this conclusion, FHL3 protein level was also significantly elevated in GC by proteogenomic analysis of Beijing dataset which contained 58 paired gastric tumor samples and non-tumor adjacent tissues (PXD011821, **Figure 1H**, *P* < 0.001). Furthermore, we explored the role of FHL3 in the progression of GC. We performed Kaplan–Meier analyses in both TCGA and KM-plotter cohorts, and the survival curves showed that patients with higher expression level of FHL3 have shorter overall survival time than those with lower expression levels in TCGA (HR = 1.40, 95% CI = 1.15–1.51, *P* = 0.039, **Figure 1I**) and KM-plotter cohorts (HR = 1.55, 95% CI = 1.31–1.84, *P* < 0.001, **Figure 1J**).

**Figure 2:**
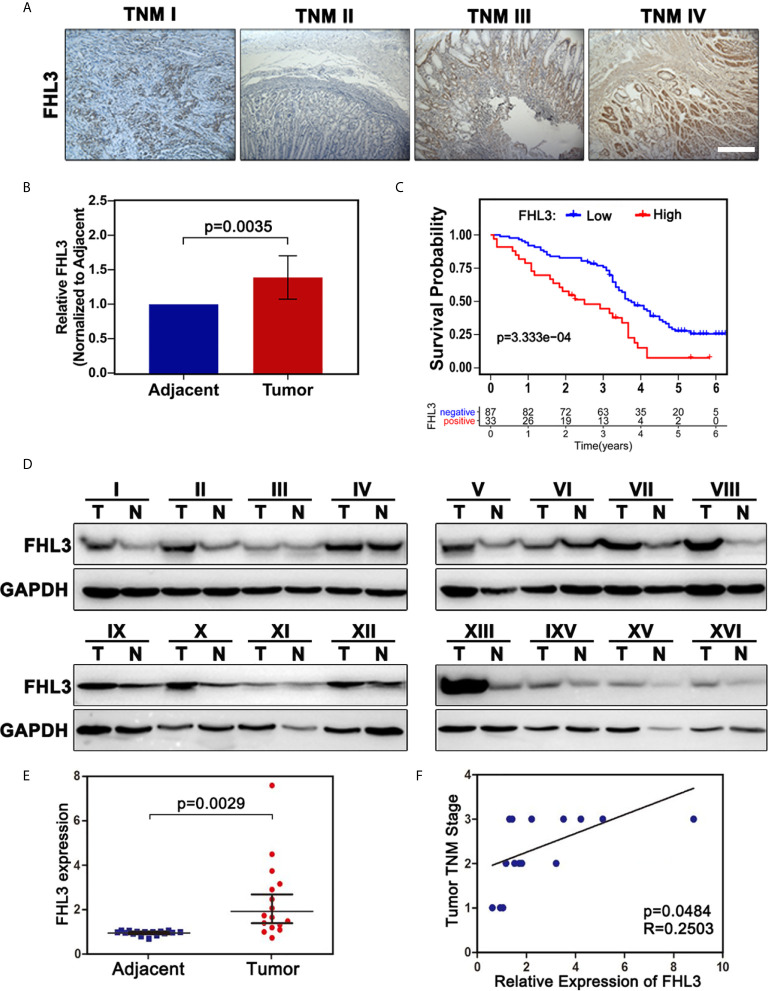
FHL3 is highly expressed in tumor samples, FHL3 expression, and associated with survival and TNM stage. The level of FHL3 in gastric cancer was analyzed in 16 fresh frozen tumor tissues and 120 paraffin-embedded sections (40× magnification). (1) FHL3 expression level in different TNM stages of gastric cancer **(A)**; (2) Relative FHL3expression level in cancer when compared with adjacent tissues **(B)**; (3) Kaplan–Meier analysis of 120 gastric cancer patients **(C)**; similar conclusion was found that higher FHL3 expression always means poor overall survival; (4) Western blot result of 16 fresh frozen tumor tissues and its adjacent tissues **(D)**; (5) Semi-quantitation analysis of western blot result of 16 fresh frozen tumor tissues and its adjacent tissues **(E)**; Relationship between FHL3 and TNM stage **(F)**.

To confirm this observation, we examined the FHL3 level in clinical gastric tumor samples. According to the IHC staining result, up-regulated FHL3 expression was positively associated with the tumor TNM stage (**Figure 2A**). Consistently, the expression of FHL3 was dramatically up-regulated in GC tissues when compared with adjacent tissues in both 120 matched paraffin-embedded tumor tissue sections and 16 paired fresh frozen tissue (**Figures 2B, D, E**, *P* < 0.05). In addition, according to the FHL3 expression and the follow-up time of 120 GC patients, Kaplan–Meier analysis indicated that higher expression of FHL3 leads to worse prognosis in GC patients (*P* < 0.001, **Figure 2C**). In 16 paired GC tissues, higher expression level of FHL3 is always accompanied with worse TNM stage (**Figure 2F**, *P* = 0.0484).

The correlations between FHL3 and clinicopathological parameters were shown in **Table 1**. There is no significant difference in FHL3 expression in different groups such as gender (*P* = 0.67), age (*P* = 0.72), tumor size (*P* = 0.50), tumor location (*P* = 0.66), depth of invasion (*P* = 0.94), and lymph node metastasis (*P* = 0.77). However, as **Table 1** shows, higher expression level of FHL3 is accompanied with differentiation (*P* = 0.009), metastasis *(P* = 0.002), and TNM stage (*P* = 0.039).

**Table 1:** Association between clinicopathological parameters and FHL3 in 120 cases of gastric cancer.

		Total patients	FHL3-positive	FHL3-negative	*P* value
Sex	Male	80	21	59	0.67
	Female	40	12	28	
Age	<60 y	50	13	37	0.72
	>60 y	70	20	50	
Tumor size	<3 cm	23	5	18	0.50
	>3 cm	97	28	69	
Differentiation	Well/moderate	101	23	78	**0.009**
	Poor	19	10	9	
Tumor location	Upper/Medium	62	17	60	0.66
	Low	58	12	31	
Depth of invasion	T1 + T2	26	7	19	0.94
	T3 + T4	94	26	68	
Lymph node metastasis	N0	24	5	19	0.77
	N1 + N2 + N3	96	28	68	
Metastasis	M0	112	27	85	**0.002**
	M1	8	6	2	
TNM stage	I + II	35	5	30	**0.039**
	III + IV	85	28	57	

Bold values mean P < 0.05.

At the same time, the prognostic values of FHL3 and clinical parameters were evaluated by using univariate and multivariate analyses, respectively. In univariate analysis, lymph node metastasis (Hazard ratio = 1.43, 95% CI: 1.15–1.78, *P* = 0.001, **Table 2**) and FHL3 (Hazard ratio = 2.29, 95% CI: 1.45–3.63, *P* < 0.001, **Table 2**) were identified as risk factors of disease progression in GC. The multivariate Cox proportional hazards model showed lymph node metastasis (Hazard ratio = 1.47, 95% CI: 1.09–1.98, *P* = .011, **Table 2**) and FHL3 (Hazard ratio = 2.06, 95% CI: 1.23–3.42, *P* = 0.005, **Table 2**) were the independent risk factors of overall survival in GC.

**Table 2:** Univariate analysis and multivariate analysis of overall survival in 120 gastric cancer patients.

Variables	Univariate analysis			Multivariate analysis		
	Hazard Ratio	95% CI	*P* value	Hazard Ratio	95% CI	*P* value
Sex	1.20	0.77–1.89	0.40	1.12	0.68–1.85	0.65
Age	0.98	0.97–1.00	0.08	0.98	0.96–1.00	**0.033**
Tumor size	1.03	0.57–1.86	0.93	0.70	0.34–1.42	0.32
Differentiation	0.85	0.58–1.25	0.41	1.20	0.72–2.00	0.49
Tumor location	0.99	0.89–1.11	0.90	0.93	0.83–1.05	0.25
Depth of invasion	1.06	0.83–1.34	0.66	1.13	0.73–1.76	0.58
Lymph node metastasis	1.43	1.15–1.78	**0.001**	1.47	1.09–1.98	**0.011**
Metastasis	1.55	0.72–3.37	0.27	1.37	0.28–6.61	0.70
TNM stage	1.32	0.95–1.83	0.10	0.97	0.47–1.99	0.94
FHL3 expression	2.29	1.45–3.63	**0.0004**	2.06	1.24–3.42	**0.005**

Bold values mean P < 0.05.

### FHL3 Knockdown Reduces OHP Resistance *In Vitro*

**Figure 3:**
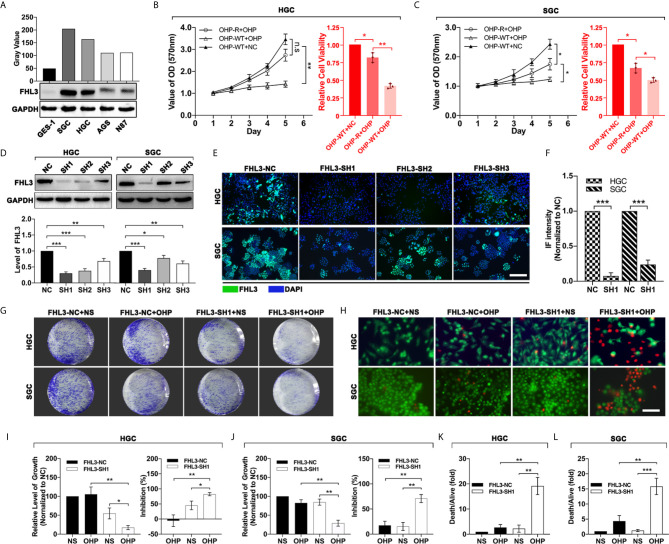
FHL3 knockdown significantly sensitized the efficacy of OHP treatment in gastric cancer. (1) The gastric cancer cells were screened for FHL3-high-expression cell lines by western blot **(A)**; (2) MMT was performed to detect the growth of OHP-resistance cell lines when treated with NS or OHP for 5 days in HGC and SGC cell lines **(B, C)**; (3) The knockdown efficiency of the four FHL3 knockdown sequences in OHP-resistance cell lines, through western blot and immunofluorescence analysis (100× magnification); we found that FHL3-SH1 was the most efficient sequence for down-regulation of FHL3 **(D–F)**; (4) OHP-resistance FHL3-NC and SH1 cell lines were treated with OHP to detect the proliferation *via* colony formation **(G, I**, **J)**; (5) OHP-resistance FHL3-NC and SH1 cell lines were treated with OHP to detect the efficacy of OHP *via* colony formation and live and death staining assay (200× magnification, **H, K** and **L**).

In order to investigate the relationship between the expression of FHL3 and chemotherapy in GC, we first screened out the FHL3-high-expression GC cell lines. We detected the FHL3 expression level in a normal gastric cell line (GES-1) and several gastric cancer cell lines (SGC, HGC, AGS and N87). As the results show, FHL3 is highly expressed in all GC cell lines in contrast with GES-1, while SGC and HGC hold the first and second highest level of FHL3 respectively (SGC-7901>HGC>AGS>N87>GES-1, **Figure 3A**). Then we established OHP-resistance (OHP-R) cell lines in SGC and HGC cell lines. As **Figures 3B, C** show, OHP-R cell lines hold higher tolerance in OHP treatment (HGC: *P* < 0.01, SGC: *P* < 0.05). Whereafter, we silenced the FHL3 by lentivirus and verified the knockdown efficiency by western blot (WB) and immunofluorescence (IF). Both WB and IF assays showed the same result that FHL3-SH1 is the most efficient group in FHL3 knockdown, and FHL3-SH1 cell line was used for our following experiments (**Figures 3D–F**). Colony formation assay and live and death staining assay were performed to detect the effect of FHL3 knockdown when gastric cancer cell received OHP therapy. In the colony formation assay, our study found that FHL3-NC cell lines have similar growth ability with the treatment of OHP and NS (**Figures 3G, I, J** **)**, while the FHL3 knockdown slightly decreased the colony unit formation (*P* < 0.05) when it received OHP therapy. Combination treatment (FHL3 knockdown and OHP) restricted the tumor growth of about 80% in OHP-R HGC/SGC cell lines (**Figures 3G, I, J**, *P* < 0.01). Collectively, FHL3 knockdown significantly enhanced the OHP-induced tumor growth inhibition rate about one-fold as compared with the single OHP treatment group (**Figures 3G, I, J**, *P* < 0.05). In the live and death staining assay, our study showed that obviously there are less live cells (green) and more death cells (red) in the combination treatment group when compared with the other groups (**Figures 3H, K, L**, P < 0.01).

### Down-Regulation of FHL3 Reverts the EMT Phenotype and Restricts Tumor Metastasis

**Figure 4:**
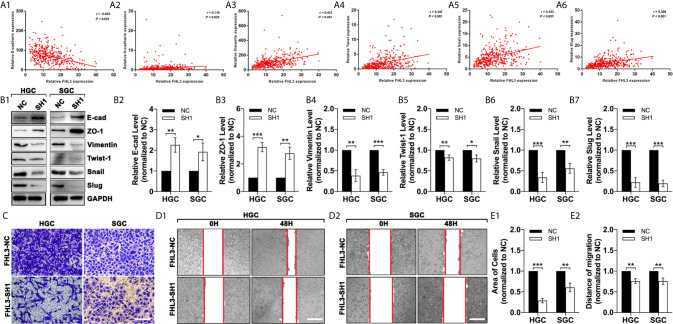
FHL3 knockdown reversed the EMT process in gastric cancer. (1) The relationship between FHL3 and EMT-associated transcriptional factors (Snail1, Slug, Twist), and EMT markers (N-cadherin, E-cadherin and Vimentin) **(A_1–6_)**; (2) FHL3 knockdown reversed the EMT process and was verified by western blot in FHL3-NC and SH1 cell lines **(B_1–7_)**; (3) The effects of FHL3 knockdown in migration and invasion were explored by transwell (200× magnification) and wound healing assay (40× magnification) in in OHP-resistance FHL3-NC and SH1 cell lines **(C, D_1–2_** and **E_1–2_)**.

To investigate the association of FHL3 and EMT, we analyzed the data in TCGA. And our study found that the expression level of FHL3 was negatively correlated to the expression level of E-cadherin (r = −0.403, *P* < 0.001, **Figure 4A1**) and positively correlated to N-cadherin (r = 0.116, *P* = 0.025, **Figure 4A2**), Vimentin (r = 0.421, *P* < 0.001, **Figure 4A3**) and Twistl (r = 0.347, *P* < 0.001, **Figure 4A4**), Snail (r = 0.353, *P* < 0.001, **Figure 4A5**) and Slug (r = 0.358, *P* < 0.001, **Figure 4A6**). These data indicated that FHL3 may contribute to EMT process. In **Figures 3B1–7**, WB showed that FHL3 knockdown significantly up-regulated the expression of E-cadherin and ZO-1 more than one-fold, while it may down-regulate the Vimentin, Slug, and Snail more than 50% (**Figures 4B1–7**, *P* < 0.01). However, Twist1 showed a slight change with the treatment of FHL3 knockdown. In the following experiments, our study finds that FHL3 knockdown restricted the cell migration more than 50% in HGC cell lines and about 50% in SGC cell lines (**Figures 4C, E1**, *P* < 0.05). In the wound healing assay, we have got a similar conclusion (**Figures 4D2, E2**).

### FHL3 Knockdown Reduces OHP Resistance and Metastasis in Subcutaneous/Orthotopic Stomach Tumor Bearing-Model and Lung Metastasis Model

**Figure 5:**
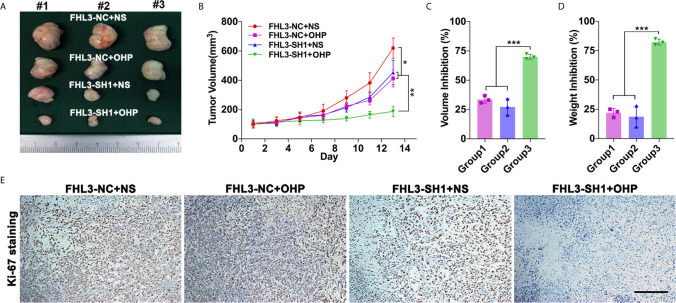
FHL3-mediated gastric cancer OHP resistance was verified in subcutaneous tumor model. (1) The tumors were harvested, and the tumor volume and weight *in vivo* were investigated **(A, C** and **D)**, FHL3 knockdown could sensitize the efficacy of OHP treatment and inhibit tumor growth; (2) Tumor volume variation during 14 days of OHP treatment **(B)**; (3) The ki-67 staining assay (100× magnification) was performed to show the cancer cell proliferation of every group **(E)**.

In *in vivo* experiment, we found the growth speed of subcutaneous tumors is slower in the single FHL3-SH1 group and OHP therapy compared with the FHL3-SH1 group (**Figures 5A, B**, *P* < 0.05). Combination treatment (FHL3-SH1 and OHP) significantly inhibits the tumor growth when compared with the FHL3-SH1 + NS and FHL3-NC + OHP groups (*P* < 0.01, **Figures 5A, B** **)**. We detected the tumor volume and weight. As **Figures 5C–D** show, combination treatment expressed better tumor growth inhibition (about one-fold) when compared with single OHP treatment or single FHL3-knockdown treatment in HGC subcutaneous tumor model (*P* < 0.001). As shown in **Figure 5E**, IHC staining was performed, and the results showed that tumors with combination treatment have a weaker intensity of Ki-67. Those results suggested FHL3-knockdown could enhance the tumor growth inhibition of OHP therapy and sensitize the chemotherapy.

**Figure 6:**
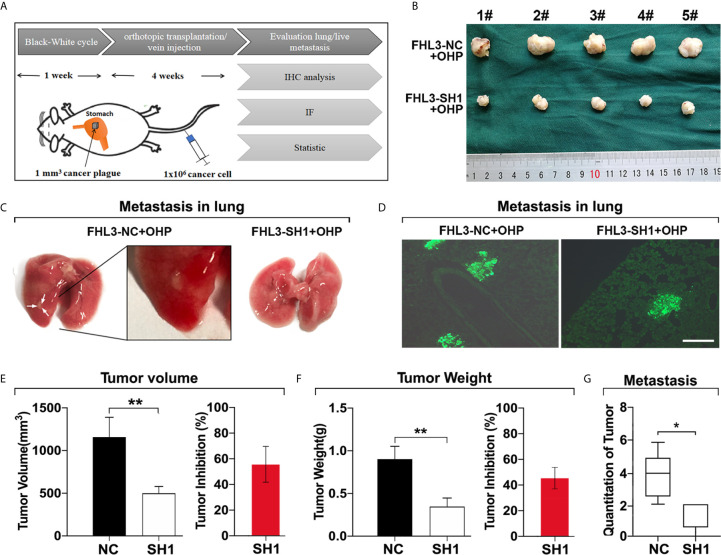
FHL3 knockdown decreased tumor metastasis in orthotopic transplantation model and lung metastasis model. (1) The schematic diagram of orthotopic transplantation model and lung metastasis model **(A)**; (2) Display of tumors harvested from orthotopic stomach after tumor transplantation and OHP treatment in SCID mice **(B, E**, **F)**; (3) Metastatic tumors in lungs were displayed in original lung specimens and the fluorescence (200× magnification) of frozen slices were detected **(C, D, G)**; those results indicated FHL3 knockdown can decrease the lung metastasis.

Then, we validated the role of FHL3 in metastasis of gastric cancer cells in vivo. As **Figure 6A** show, the orthotopic tumor was performed, and tail intravenous injection of gastric cancer cells was done. 4 weeks later, the orthotopic stomach tumors and lung metastasis nodes were harvested for tumor detection. In this section, we found orthotopically transplanted tumors FHL3-SH1 treated with OHP were more than 50% smaller, when compared with FHL3-NC treated with OHP (P < 0.001, **Figures 6B, E, F**). According to the white nodules on the surface of the lungs (**Figure 6C**), we could easily found the metastasis in situ in mice of the FHL3-NC + OHP group. We detected slices from these lung samples and performed immunofluorescence (**Figures 6D, G**), the green area can be identified as metastatic tumor from the blood cycle in the lung (which may come from tail vein injection or orthotopic stomach in situ). According to our observation, lung metastasis occurred in 1/5 SCID mouse in the FHL3-SH1 + OHP group (20%), while it occurs in 4/5 SCID mice in FHL3-NC + OHP (80%). Those results indicated FHL3 knockdown can improve the OHP efficacy and decrease the lung metastasis more than 60%.

### Down-Regulated TGF-*β*/Smad-Independent Pathways Are Features of FHL3-Knockdown-Induced Mesenchymal–Epithelial Transition

**Figure 7:**
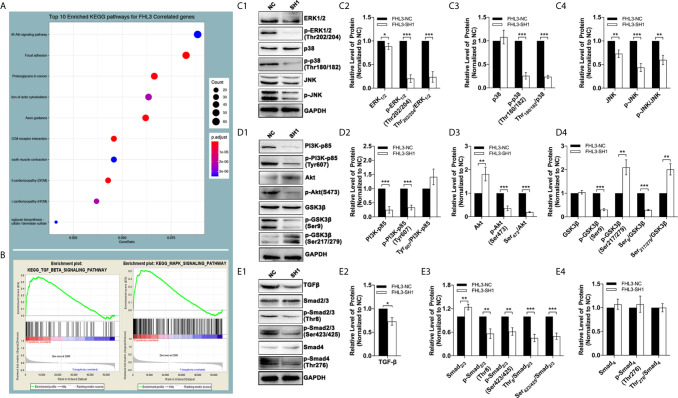
The genetic characteristics and pathways’ activation by FHL3 knockdown. (1) In KEEG and GO analyses, MAPK, PI3K/Akt and TGF*β* pathways are related to FHL3 **(A, B)**; (2) The expression and activation levels of MAPK pathway proteins were detected by western blot: down-regulation of ERK1/2, Thr202/204-ERK_1/2_ (p-ERK_1/2_), p-ERK_1/2_/ERK_1/2_ and Thr180/182-P38 and p-P38/P38 by FHL3 knockdown was observed **(C_1–4_)**; (3) The expression and activation levels of PI3K pathway proteins were detected: down-regulation of PI3K-p85, Tyr607-PI3K-p85 (p-PI3K), S473-Akt (p-Akt), p-Akt/Akt, Ser9-GSK3*β* and Ser9-GSK3*β*/GSK3*β*, and up-regulation of Akt, Ser217/279-GSK3*β* and Ser217/279-GSK3*β*/GSK3*β* by FHL3 knockdown were observed **(D_1–4_)**; (4) The expression and activation levels of TGF*β* pathway proteins were detected: down-regulation of TGF*β*, Thr8-Smad_2/3_ (p-Smad_2/3_), Ser423/425-Smad_2/3_ (p-Smad_2/3_), and p-Smad_2/3_/Smad_2/3_ by FHL3 knockdown were observed, while the status of Smad_4_ was not significantly affected by FHL3 **(E_1–4_)**.

In this section, we explored the mechanisms of FHL3-mediated EMT and EMT-associated OHP resistance in GC. The results of bioinformatic analysis revealed that the PI3K–Akt pathway, Cytoskeleton, focal adhesion, and ECM (extracellular matrix) play important roles in GC tumorigenesis and progression, which are dramatically correlated with FHL3 expression (**Figure 7A**). Moreover, as the GSEA results have shown, TGF*β* and MAPK signaling pathways tend to be significantly activated in patients with higher expression level of FHL3 (**Figures 7B**, *P* < 0.05). FHL3 may promote EMT-mediated tumor invasion and EMT-associated chemotherapy resistance in GC progression through various pathways. In order to verify our hypothesis, we detected the protein level changes of TGF*β*/Smad-independent and dependent pathways during the FHL3-knockdown-induced EMT-reversed process. As **Figures 6C1–4** have shown, FHL3 knockdown slightly decreased the level of Extracellular-regulated Kinase 1/2(ERK1/2), but greatly down-regulated the level of phosphorylated ERK_1/2_ (Thr202/204-ERK_1/2_, named p-ERK_1/2_ here) more than 50% (*P* < 0.001). And the ratio of p-ERK_1/2_/ERK_1/2_ is also down-regulated by FHL3 knockdown (**Figures 7C1–2**, *P* < 0.001). In addition, FHL3 knockdown also decreased the level both of Thr180/182-P38 (p-P38) and phosphorylated JNK (p-JNK) more than 50% (**Figures 7C1, 3, 4**, *P* < 0.001). The ratio of p-P38/P38 and p-JNK/JNK is both significantly down-regulated (**Figures 7C1, 3, 4**, *P* < 0.01). Those results implied that down-regulation of FHL3 could lead to down-regulated activity of MAPK/ERK|JNK|P38 pathways during FHL3-knockdown-induced MET process. Moreover, FHL3 knockdown also decreased the level of PI3K-p85 and Tyr607-PI3K-p85 (p-PI3K) more than 50%, while the ratio of p-PI3K/PI3K has not changed too much (**Figures 7D1–2**, *P* > 0.05). At the downstream of PI3K, Ser473-Akt (p-Akt) was also significantly down-regulated more than 50% by FHL3 knockdown, while the level of Akt was up-regulated about 1.7-fold (**Figures 7D1, 3**, *P* < 0.01). However, the ratio of p-Akt/Akt was still greatly down-regulated by FHL3 knockdown (**Figures 7D1, 3**, *P* < 0.001). Those results implied FHL3-knockdown induced down-regulated activity of PI3K and up-regulated activity of Akt. Then we found those changes may accompany with the down-regulation of Ser9-GSK3*β* and Ser9-GSK3*β*/GSK3*β*, and the up-regulation of Ser217/279-GSK3*β* and Ser217/279-GSK3*β*/GSK3*β* (**Figures 7D1, 4**, *P* < 0.01). According to those results, FHL3 knockdown inhibited the activity of PI3K/Akt pathway, leading to up-regulated activity of GSK3*β*. Collectively, the activities of TGF*β*/Smad-independent pathways were significantly regulated during the FHL3-induced EMT.

To the best of our knowledge, for TGF*β*/Smad-dependent way, Smad complex directly regulates the expression level of EMT-TFs to promote EMT process. However, in our experiments, FHL3 knockdown contributed no effect on the regulation of Smad4 and p-Smad4, while it exactly decreased the level of TGF*β*, Thr8-Smad2/3 (p-Smad2/3), Ser423/425-Smad2/3 (p-Smad2/3), and p-Smad2/3/Smad2/3. In other words, FHL3 knockdown has no effect on the regulation of TGF*β*/Smad-dependent pathway (**Figures 7E1–4**).

### RNF146-Mediated Degradation Participates in FHL3-Induced EMT Process

**Figure 8:**
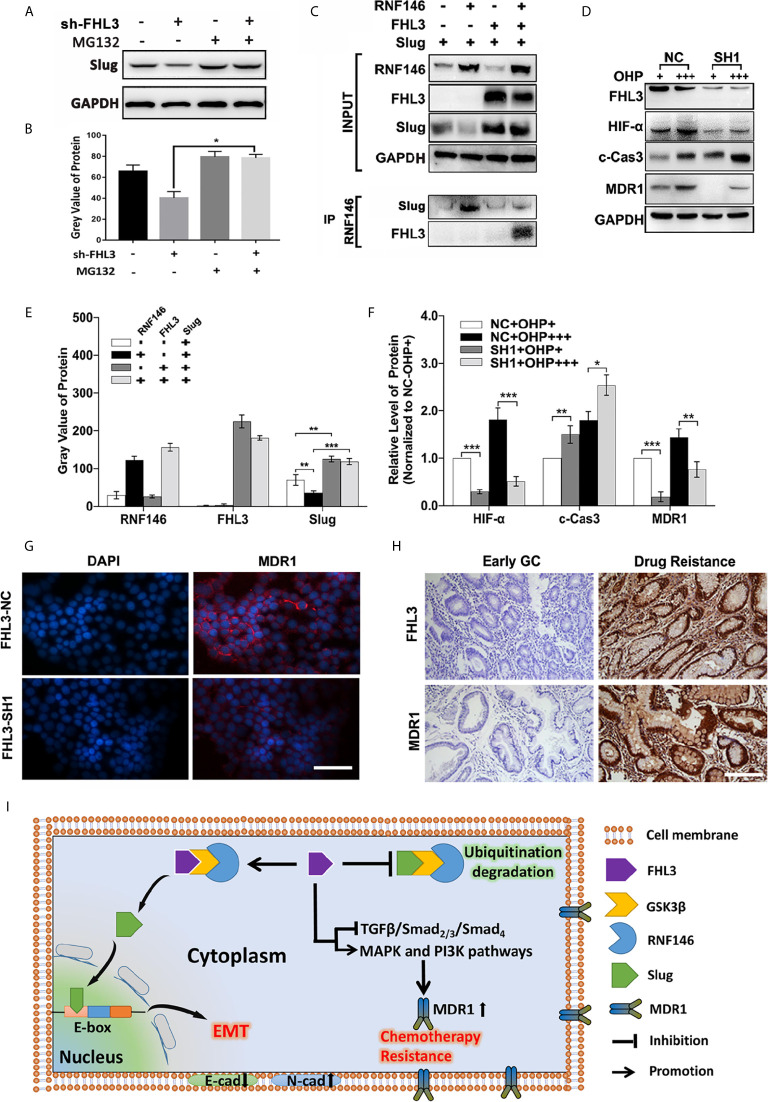
The molecular mechanisms of FHL3-mediated EMT and chemotherapy resistance. (1) Ubiquitin inhibitor bortezomib was added into the medium of FHL3-NC and SH1 to observe the regulation of Slug; it might reverse the FHL3-downregulation-induced decrease of Slug **(A, B)**; (2) 293T cell was transfected with Slug-plasmid and/or RNF146-plasmid and FHL3-plasmid, and the basic expression of those protein was detected by western blot **(C, E)**; Co-IP assay was used to detect the interaction between FHL3, Slug, and RNF146, and the result suggested that RNF146-myc can interact with FHL3-HA and Slug-Flag **(C)**; (3) The levels of HIF-1*α*, cleaved-caspase-3 and MDR1 were investigated in OHP-resistance FHL3-NC/SH1cells **(D, F)**; (4) The relationship of FHL3 and MDR1was investigated in GC cell line and slices; IHC showed that advanced GC with chemotherapy resistance has higher level of FHL3 and MDR1 (200× magnification) **(G, H)**. (5) Mechanism scheme: FHL3 leads to EMT *via* competitively bonding the ubiquitin complex with Slug, and multi-pathway activation contributes to chemotherapy resistance **(I)**.

In our previous study, we have found that FHL3 participates in Akt/GSK3*β* pathway-mediated ubiquitination degradation of EMT-TFs, and the E3 ligase RNF146 was firstly reported to interact with FHL3 in pancreatic cancer. Firstly, we treated the NC and sh-FHL3 GC cells with MG132 (an ubiquitin inhibitor) (5 uM, 3 h), and we found that when FHL3 was knocked down, the MG132 can reverse the degradation of Slug, and the protein level of Slug was up-regulated (**Figures 8A, B** **)**. Then, we explored the role of RNF146 in FHL3-mediated stabilization of EMT-TFs in GC. As **Figure 8C** showed, overexpression of RNF146 restricted the pcDNA-Slug recombination-plasmid-mediated up-regulation of Slug about 50% (**Figures 8C, E**, *P* < 0.01). In addition, overexpression of FHL3 significantly enhanced pcDNA-Slug recombination-plasmid-mediated up-regulation of Slug (**Figures 8C, E**, *P* < 0.01). Furthermore, FHL3 overexpression eliminated the RNF146 overexpression-mediated down-regulation of Slug (**Figures 8C, E**, *P* < 0.01). Then the CO-IP assay shows that RNF146 can interact both with FHL3 and Slug (**Figure 8C**). Those results implied that RNF146 participates in the ubiquitination degradation of Slug; FHL3 may interfere with EMT process by competitively bonding the ubiquitin complex with Slug.

### FHL3 Induces Chemoresistance *via* MDR1

According to previous studies, TGF*β*/Smad-independent pathways containing MAPK/ERK and PI3K/Akt participated in chemotherapy resistance, and those pathways have been pointed out to be probably associated with OHP resistance in GC. However, more details are needed to figure out the mechanism of FHL3-mediated chemotherapy resistance. So, we detected the level of Hypoxia-Inducible Factor 1-*α* (HIF-*α*), caspase-3, and MDR1 in FHL3-knockdown cell lines. The results showed that high-dose treatment of OHP led to up-regulation of HIF-*α* about 0.2-fold, caspase-3 about 0.7-fold, and MDR1 about 0.4-fold in OHP-R FHL3-NC HGC cells, respectively (**Figures 8D, F**, *P* < 0.05). FHL3 knockdown restricted the high-dose treatment of OHP-induced up-regulation of HIF-*α* and MDR1 at least 50%, while it up-regulated the level of caspase-3 about 0.25-fold (**Figure 8D**, *P* < 0.05). Then, we explored the association of FHL3 and MDR1 *via* detecting the expression through IF and IHC. The IF showed that FHL3-NC cells could have higher level of MDR1and FHL3, and knockdown of FHL3 could decrease the expression of MDR1 (**Figure 8G**). The IHC showed that advanced GC samples with chemotherapy resistance have higher level of FHL3 and MDR1 when compared with early GC (**Figure 8H**).

## Discussion

Although the morbidity and mortality of GC have declined over the past decade, we still face many problems and challenges in the screening and treatment of GC. TNM stage, encompassing the depth of invasion (T), lymph node metastasis (N), and distant metastasis (M) stages were regarded as the most significant prognostic factors of GC. Due to tumor heterogeneity, even GC patients with the same TNM stage may have different prognoses in survival time after complete surgical resection, indicating that prognosis cannot be accurate if we determined it based on the current staging system. Even receiving complete resection or targeted therapy, many advanced GC patients still die of local recurrence and/or distant metastasis, among which tumor metastasis and chemotherapy failure are the severe problems for all clinical doctors. Although various genes and pathways have been investigated in GC, the mechanisms of metastasis and chemoresistance are still unclear.

Recently, LIM domain-only protein family plays pivotal roles in tumor progression, including radiotherapy resistance and metastasis (7, 28). Some studies showed that FHLs play as a tumor repressor in breast cancer, liver cancer, and lung cancer (32–34), while other studies pointed out that FHLs promote paclitaxel resistance and radiotherapy resistance in liver cancer and HeLa cell respectively (28, 35), enhance tumor cell growth in liver cancer, glioma, and breast cancer (36, 37), and lead to metastasis in breast cancer and pancreatic cancer (7, 30). So far, the role of FHLs in gastric cancer is still unclear. In our study, we found the expression level of FHL3 was obviously up-regulated in GC both in mRNA and protein by analysis in TCGA, GTEx, and Beijing dataset (*P* < 0.05, **Figures 1A–H**). Meantime, the same results were obtained from 16 fresh frozen tumor tissues and 120 paraffin-embedded sections (*P* < 0.01). Then, 120 samples showed higher level of FHL3, leading to lower differentiation (*P* = 0.009, **Table 1**), metastasis trend *(P* = 0.002, **Table 1**) and worse stage of TNM (*P* = 0.039, **Table 1**) in GC. Besides, FHL3 was negatively associated with the prognosis in GC through Kaplan–Meier analysis in TCGA, KM-plotter cohorts and 120 GC samples (*P* < 0.05, **Figures 1I, J**, **2C**). Furthermore, the univariate analysis showed higher level of FHL3 can accompany higher risk of GC progression (**Table 2**, HR = 2.06, *P* = 0.005). In other words, those results suggested that FHL3 was a potential predictor of disease progression and prognostic factor in GC.

In the following experiments, the tumor growth was decreased ~50% in HGC and ~20% in SGC by the FHL3 knockdown (**Figures 3G, I, J**). In subcutaneous tumor model, our study found that FHL3 knockdown reduced the tumor growth ~25% of tumor volume and ~25% of tumor weight (**Figures 5C, D** **)**. Besides, FHL3 knockdown enhanced the efficacy of OHP both in HGC and SGC (**Figures 3G, I, J** **)**, which was similar to the results in subcutaneous tumor model (**Figures 5A–D**). In bioinformatic analysis, we found the level of FHL3 was positively related to the level of Slug, Snail, Twist1, N-cadherin and Vimentin, while it was negatively related to the level of E-cadherin (**Figures 4A1–6**). FHL3 knockdown obviously decreased the expression level of Vimentin, Snail, and Slug and increased the level of E-cadherin and ZO-1 both in HGC and SGC (**Figures 4B1–7**). Meantime, migration ability was reduced by FHL3 knockdown (**Figures 4C–E2**).

The mechanisms of how FHL3 regulates tumor chemotherapy resistance and metastasis were unclear in GC. To our knowledge, EMT was mainly responsible for tumor metastasis, and the lower level of E-cadherin was considered as the feature in the EMT process (6, 9). E-box is a pivotal DNA reading frame in CDH1 sequence through which some transcriptional factors can directly bind to CDH1 to regulate the expression level of E-cadherin, such as Snail1/2, Twist1/2, Zeb1, and FOXC2 (9). Besides, other cellular junction proteins also play roles in tumor metastasis, such as N-cadherin and ZO-1. The regulation of EMT holds some recognized pathways, among which the TGF*β*-mediated Smad-dependent and Smad-independent ways were the major roles (6, 9). For TGF*β*/Smad-dependent way, Smad complex directly regulates the expression level of EMT-TFs to promote the EMT process (6, 9). Previous studies have shown that FHLs promoted phosphorylation of Smad_2/3_ and directly interacted with them, leading to nuclear accumulation of Smad complex in liver cancer (32). For TGF*β*/Smad-independent way, PI3K/Akt, MAPK, Nuclear Factor Kappa-B (NF-*κ*B), Hedgehog, and Wnt/β-catenin pathways were upstream regulators of EMT-TFs (38–41). Some studies pointed out that FHLs can increase the activation and transcription of Akt to promote tumor growth and progression in glioma, breast cancer, and ovarian cancer (30, 42, 43). Other published papers showed that FHLs interfere with the MAPK/ERK pathway, resulting in radiotherapy resistance in pancreatic cancer (44). It was reported that FHL knockdown contributed to tumorigenesis prevention in osteosarcoma by down-regulation of Wnt/*β*-catenin pathways (27). FHLs were also associated with hepatocarcinogenesis by activating NF-*κ*B pathway (45). Therefore, FHL3 was likely to regulate gastric cancer metastasis through TGF*β*/Smad-independent pathway. Encouragingly, our study found MAPK, PI3K/Akt, and TGFβ pathways were close to FHL3 by KEEG and GO analysis (**Figures 5A, B**). And in the following experiments, FHL3 knockdown obviously down-regulated the phosphorylation level of MAPK pathway downstream molecules ERK1/2, P38, and JNK in HCG (**Figures 5C1–4**). Besides, FHL3 knockdown obviously down-regulated the phosphorylation level of PI3K, Akt, and GSK3*β* in HCG (**Figures 5D1–4**). However, FHL3-knockdown-induced down-regulation of TGF*β* has few effects on the phosphorylation level of Smad4. Collectively, FHL3-induced EMT was associated with the activation of MAPK/ERK|JNK|P38 and PI3K/Akt/GSK3*β* pathways. Furthermore, based on our previous study that FHL3 regulated the Akt/GSK3*β*/ubiquitin-Snail1|Twist1 pathway to stabilize the EMT-TFs to promote EMT process in pancreatic cancer, we explored the role of FHL3 in the regulation of ubiquitin-mediated EMT. Here, we hypothesized that E3 ligase RNF146 can form a complex with FHL3 and Slug *via* GSK3β, and the higher level of FHL3 may induce up-regulation of Slug *via* inhibiting the degradation (**Figures 8A, B** **)**.

The mechanism of chemotherapy resistance is complicated. ABC transporters, especially for MDR1, had a central role in chemo-drug efflux to make chemotherapy resistance in gastric cancer (46, 47). MDR1 can be directly regulated by the NF-*κ*B pathway. Hypoxia also participated in the regulation of MDR1; up-regulation of HIF-*α* can lead to chemotherapy in gastric cancer (23). Previous studies showed that inhibition of apoptosis was also important for chemotherapy, and the apoptosis-inhibition-mediated chemotherapy was regulated by MAPK pathway and PI3K/Akt pathway (20–22). Besides, EMT was also considered to be responsible for chemotherapy resistance in pancreatic cancer and breast cancer (12, 13). Nevertheless, the characteristic changes of some signaling pathways in mesenchymal phenotype may be related to the chemotherapy resistance. Up-regulation of EMT-TFs, such as Snail and Twist1, simultaneously increased the level of ABC transporters to endow chemotherapy resistance during its up-regulation of the EMT process (17–19). In fact, our study found that down-regulation of FHL3 promoted the mesenchymal–epithelial transition (MET), during which it may reduce chemotherapy resistance in HGC. Furthermore, our study found MDR1 was down-regulated by FHL3 knockdown (**Figures 8D, E** **)**. In conclusion, those TGF*β*/Smad-independent pathways were the regulators in FHL3-mediated chemotherapy resistance.

Collectively, as the scheme of our hypothesis showed (**Figure 8I**), FHL3 could competitively bond the complex (GSK3β/RNF146) with Slug. Slug could induce the EMT process and promote cancer cell metastasis. In addition, FHL3 could induce drug resistance by activating the MAPK and PI3K pathway which may lead to the MDR1 overexpression.

## Data Availability Statement

The datasets presented in this study can be found in online repositories. The names of the repository/repositories and accession number(s) can be found in the article/**Supplementary Material**.

## Ethics Statement

The studies involving human participants were reviewed and approved by the First Affiliated Hospital of Anhui Medical University Review Board and the ethics committees of Anhui Medical University. The patients/participants provided their written informed consent to participate in this study. The animal study was reviewed and approved by the First Affiliated Hospital of Anhui Medical University Review Board and the ethics committees of Anhui Medical University. Written informed consent was obtained from the individual(s) for the publication of any potentially identifiable images or data included in this article.

## Author Contributions

GC,PL and XH contributed equally to this work. Software: MJ. Resources: SC and XX. Writing: GC. Conceptualization: QS, MX and BC. All authors contributed to the article and approved the submitted version.

## Funding

This study is supported by Key Research and Development Plan Projects of Anhui Province (Project Nos.201904a07020045); Quality Engineering Projects of Anhui Province (No: 2020jyxm0898; No: 2020jyxm0910; No: 2019kfkc334); Clinical research project of Anhui Medical University (No:2020xkj176).

## Supplementary Material

The Supplementary Material for this article can be found online at: https://www.frontiersin.org/articles/10.3389/fonc.2021.649029/full#supplementary-material

## Conflict of Interest

The authors declare that the research was conducted in the absence of any commercial or financial relationships that could be construed as a potential conflict of interest.
