# Supplementary figures and images for: FHL3 Contributes to EMT and Chemotherapy Resistance Through Up-Regulation of Slug and Activation of TGFβ/Smad-Independent Pathways in Gastric Cancer
Source: Front Oncol. 2021 Jun 4;11:649029. doi: 10.3389/fonc.2021.649029 (PMC8213027; doi:10.3389/fonc.2021.649029)

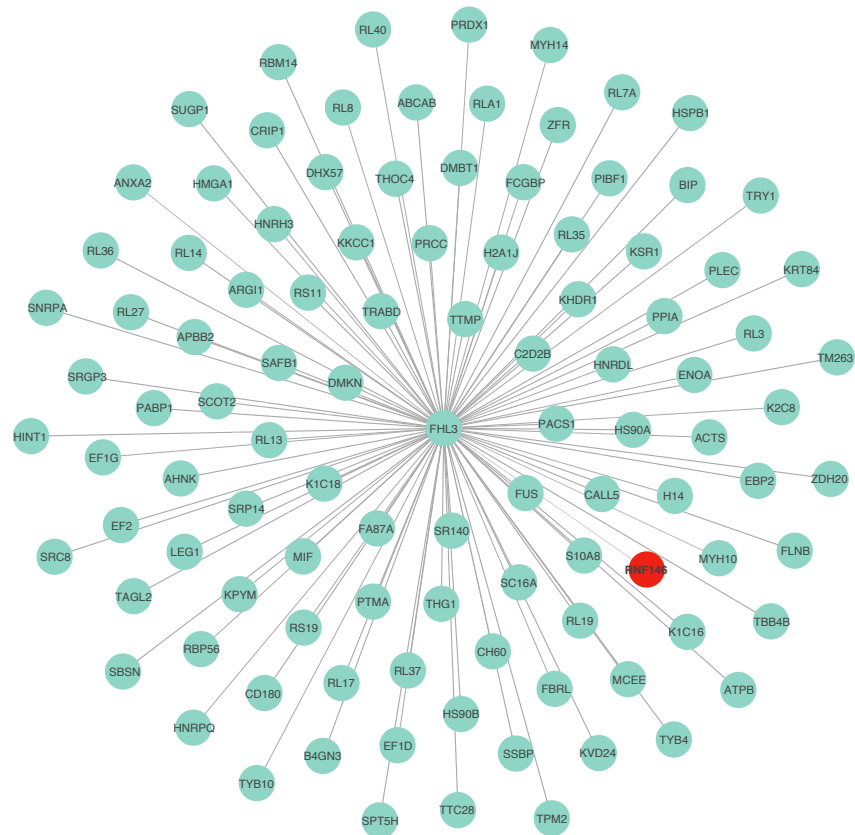

Supplement: Supplementary file 1 [file Image_1.pdf]
